# Targeting the Hippo Pathway Is a New Potential Therapeutic Modality for Malignant Mesothelioma

**DOI:** 10.3390/cancers10040090

**Published:** 2018-03-22

**Authors:** Yoshitaka Sekido

**Affiliations:** 1 Division of Molecular Oncology, Aichi Cancer Center Research Institute, 1-1 Kanokoden, Chikusa-ku, Nagoya 464-8681, Japan; ysekido@aichi-cc.jp; Tel.: +81-52-764-2983; 2 Department of Cancer Genetics, Nagoya University Graduate School of Medicine, Nagoya 464-8681, Japan

**Keywords:** malignant mesothelioma, NF2, Hippo pathway, YAP1/TAZ

## Abstract

Malignant mesothelioma (MM) constitutes a very aggressive tumor that arises from the pleural or peritoneal cavities and is highly refractory to conventional therapies. Several key genetic alterations are associated with the development and progression of MM including mutations of the *CDKN2A/ARF*, *NF2*, and *BAP1* tumor-suppressor genes. Notably, activating oncogene mutations are very rare; thus, it is difficult to develop effective inhibitors to treat MM. The *NF2* gene encodes merlin, a protein that regulates multiple cell-signaling cascades including the Hippo pathway. MMs also exhibit inactivation of Hippo pathway components including LATS1/2, strongly suggesting that merlin-Hippo pathway dysregulation plays a key role in the development and progression of MM. Furthermore, Hippo pathway inactivation has been shown to result in constitutive activation of the YAP1/TAZ transcriptional coactivators, thereby conferring malignant phenotypes to mesothelial cells. Critical YAP1/TAZ target genes, including prooncogenic *CCDN1* and *CTGF*, have also been shown to enhance the malignant phenotypes of MM cells. Together, these data indicate the Hippo pathway as a therapeutic target for the treatment of MM, and support the development of new strategies to effectively target the activation status of YAP1/TAZ as a promising therapeutic modality for this formidable disease.

## 1. Introduction

Malignant mesothelioma (MM) constitutes an aggressive neoplasm that arises primarily from pleura or peritoneum serosal cells, such that almost 80% of cases are pleural in origin [1,2]. However, current radiological diagnostic methods for MM, which consist of chest X-rays and computed tomography (CT) scans, are not effective at detecting malignant pleural mesothelioma (MPM) during the early stages of disease progression. Furthermore, despite continuous efforts to develop more sensitive and specific MM blood biomarkers, such as the recent demonstration of elevated high mobility group box protein 1 (HMGB1) levels in the serum of patients with MM [3], suitable blood or pleural effusion biomarkers for this disease have not yet been established [4]. Thus, MM is usually diagnosed at an advanced stage. In addition, as the anatomical characteristics of the body cavities where MM initially develops enable MM cells to easily disseminate via body fluids, it is also very difficult to completely surgically remove MM tumors without leaving residual microscopic disease. 

MMs are pathologically classified as either epithelioid, sarcomatoid, or biphasic (i.e., consisting of both epithelioid and sarcomatoid components) [5]. As they are often refractory to conventional therapies, they often incur a poor patient prognosis, with the median overall survival (OS) time for patients with MPM being 12–18 months after diagnosis, regardless of trimodality therapy consisting of induction chemotherapy followed by surgery and post-operative radiation therapy [6]. The expected OS time is reduced for patients with non-epithelioid (i.e., sarcomatoid or biphasic) compared to epithelioid MM [7]; likewise, patients that exhibit an advanced overall tumor stage, higher T or N category, Eastern Cooperative Oncology Group (ECOG) performance status of 1 or higher, and/or are male also experience a worse overall prognosis [7]. 

Recent investigations into the genetic factors that underlie MM pathogenesis have identified frequent mutations in *CDKN2A/ARF*, *NF2*, BRCA1-associated protein 1 *(BAP1)*, and *TP53*, albeit only very rare mutations in other well-known oncogenes [8]. A relatively small number of somatic mutations have also been observed in MM cases, whereas chromosome loss and/or mutations that affect genes involved in histone modification (*SETDB1* and *SETD2*) and RNA processing (*DDX3X*, *DDX51*, and *SF3B1*), appear to be more common [8]. Epidemiologically, MM is most often caused by exposure to asbestos, with onset occurring after a long period of latency [9]. Notably, however, it can also be caused by a familial cancer syndrome arising from germline *BAP1* mutations [10].

## 2. Current Standard, and ‘Under-Clinical-Trial’ Therapeutic Modalities for MM

Owing to its characteristic diffuse growth pattern and the resultant lack of surgical margins, it is theoretically impossible to achieve complete microscopic resection of MPM. Thus, two types of curative (intent) surgery are currently available to patients; extrapleural pneumonectomy (EPP) and pleurectomy/decortication (P/D). Maximal surgical cytoreduction treatments for MPM are currently performed in combination with chemotherapy, with or without radiation therapy [11].

Only two chemotherapy drugs, cisplatin, and the anti-folate drug pemetrexed, are currently approved and used as part of the first-line regimen for patients with advanced MM. Notably, administering a combination of these drugs alone has only been shown to slightly increase patient OS [12]. In addition, although novel molecularly targeted drugs have been recently shown to stabilize MM disease progression, none are currently recommended as standard MM treatments [13]. 

The first-generation tyrosine kinase inhibitors erlotinib and gefitinib, which target the epidermal growth factor receptor (EGFR), were shown not display any significant activity in MM cases [14]. Similarly, the multi-targeted small-molecule tyrosine kinase inhibitors cediranib, dasatinib, sorafenib, and sunitinib each failed to show adequate clinical activity as second-line treatments when administered as monotherapies [15,16,17,18]. In contrast, a recent phase-II trial found that an angiokinase inhibitor termed nintedanib, which targets vascular endothelial growth factor receptors (VEGFRs), platelet-derived growth factor receptors (PDGFRs), fibroblast growth factor receptors (FGFRs), and Src and Abl-kinase signaling, improved the progression free survival (PFS) time for patients with MPM when administered in combination with pemetrexed and cisplatin [19]. This effect is currently being confirmed via an ongoing phase-III trial [19]. Another phase-III study recently showed that administering bevacizumab (Avastin^®^, Genentech, South San Francisco, CA, USA), a humanized anti-VEGF antibody, in combination with pemetrexed and cisplatin significantly increased patient OS [20]. However, further investigation of this effect was halted in 2017 to allow the drug manufacturer the opportunity to seek approval from global health authorities to pioneer Avastin^®^ as a treatment for MPM. 

Immune checkpoint inhibitors including anti-CTLA4 (tremelimumab and ipilimumab), anti-PD1 antibodies (nivolumab and pembrolizumab), and anti-PD-L1 antibodies (avelumab and durvalumab) are currently undergoing intensive investigation in relevant MM clinical trials [21,22,23]. Thus far, tremelimumab treatment has been shown to not significantly prolong the OS of patients previously treated for MM compared to placebo [22]. Although the definitive conclusions of the anti-PD1/PD-L1 antibody studies have not yet been reported, administering immune checkpoint anti-PD1 or anti-PD-L1 antibodies either alone or in combination with the alternative-type inhibitor such as an CTLA-4 antibody appears to confer some benefits to a subset of patients with MM [23]. Thus, a combination of different types of immune checkpoint inhibitors may elicit a better patient response to treatment; although notably, the incurred side-effects may also be exacerbated, and may therefore require careful management.

Epigenetic MM therapies have also been tested [24]; however, the DNA methyl transferase (DNMT) inhibitor dihydro-5-azacytidine, and the histone deacetylase (HDAC) inhibitors vorinostat and belinostat showed only a modest [25], and no clinical effects [14,26], respectively. As BAP1 loss has been found to increase both the activity of EZH2 (which is a component of the polycomb repressor complex 2 (PRC-2)) and the levels of trimethylated histone H3 lysine 27 (H3K27me3), a recent study assessed the effects of EZH2 inhibition on MM progression. The results of this study demonstrated that inhibiting EZH2 suppressed the proliferation of *BAP1*-mutant MM cell lines, suggesting that EZH2 inhibitors may represent promising candidate therapeutic agents for MM [27]. 

Other promising antitumor agents include monoclonal antibody-based drugs against mesothelin [28] and CD26 [29]. MMs often exhibit reduced expression of arginosuccinate synthetase-1 (ASS1); thus, a synthetic lethal approach with pegylated-arginine deiminase ADI-PEG20, which depletes available arginine, showed a significant PFS improvement for patients with MM [30]. Additional novel MM treatment strategies based on alterations of tumor suppressor genes, including *BAP1,* are currently under development [31,32]. 

In terms of radiotherapy, intensity-modulated radiation therapy (IMRT) has been shown to potentially confer a survival benefit to a subset of patients with MM [33]. In addition, novel innovative approaches with pleural and induction-accelerated hemithoracic radiotherapy followed by surgery have been recently shown to be both feasible and safe [33]. Accordingly, the most recent American Society of Clinical Oncology Clinical Practice Guidelines provided evidence-based recommendations for the diagnosis and staging of patients with MPM, as well as for the subsequent administration of chemotherapeutic, surgical cytoreduction, radiation, and/or multimodal therapies [34].

## 3. Hippo Pathway Dysregulation in MM Cells

### 3.1. NF2 and the Hippo Pathway

**Figure 1:**
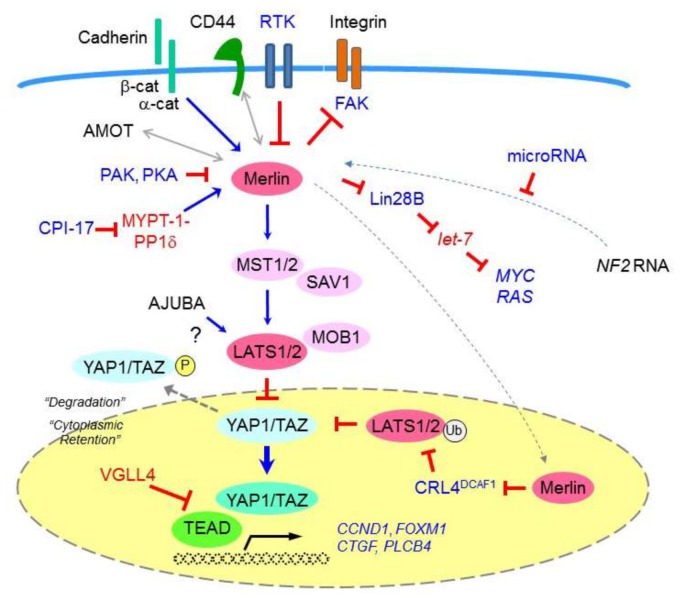
Schematic representation showing *NF2*/merlin-Hippo signaling cascade dysregulation in malignant mesothelioma (MM) cells. Signals from the extracellular environment, transduced via cell-cell contact (cadherin), cell-matrix contact (CD44 and integrin), and/or growth factor receptors (receptor tyrosine kinases, RTKs), affect merlin tumor-suppressive activity. Activated (underphosphorylated) merlin regulates the Hippo signaling cascade and suppresses the activity of the YAP1/TAZ transcriptional coactivators. As MMs show frequent alteration of merlin (the *NF2* gene product) and Hippo pathway components including LATS1/2, the resultant underphosphorylated (activated) YAP1/TAZ transcriptional coactivators enhance the expression of many pro-oncogenic genes including *CCDN1*, *FOXM1*, *CTGF*, and *PLCB4* in MM cells. P: phosphorylation, Ub: ubiquitination.

The neurofibromatosis type 2 (*NF2*) gene encodes the tumor suppressor protein moesin-ezrin-radixin-like protein (merlin), which is a member of the Band 4.1 family of cytoskeletal linker proteins [35] (Figure 1). One of the important downstream signaling cascades that are regulated by merlin is the Hippo pathway, which is involved in critical biological processes including organ-size control, development, differentiation, tissue regeneration (via cell-growth restriction), cell division regulation, apoptosis, and cancer development [36,37]. The four core components in this pathway comprise MST1 and MST2 kinases, SAV1 (also termed WW45), MOB1, and LATS1 and LATS2 kinases, all of which have been shown to act as tumor suppressors (Figure 1). Upon receiving upstream signaling, MST1/2 kinases (in a complex formed with the SAV1 scaffold protein) phosphorylate and activate LATS1/2. The latter is activated by the MOB1 scaffold protein to then phosphorylate and inactivate the YAP1 and TAZ transcriptional coactivators. The phosphorylated YAP1/TAZ are then directed to either be retained within the cytoplasm (via an interaction with 14-3-3) or degraded (via poly-ubiquitination). Conversely, when the Hippo pathway is not active, underphosphorylated YAP1/TAZ enter the nucleus and act as transcriptional coactivators. YAP1/TAZ interact with several distinct transcription factors including TEA domain (TEAD) transcription factors, SMADs, p73, Runt related transcription factor (RUNX), T-Box 5 (TBX5), the carboxyl-terminal fragment of Erb-B2 receptor tyrosine kinase 4 (ERBB4), and early growth response-1 (EGR1) [38].

Merlin also activates the Hippo pathway and suppresses YAP1/TAZ via its effects on cell junction-associated proteins known to modulate Hippo signaling [39]. Specifically (in addition to other ERM proteins and the intracellular CD44 domain), merlin binds to α-catenin and angiomotin at maturing adherens and tight junctions, respectively, to suppress cell growth. Although the relationship between angiomotin and merlin in the Hippo pathway is not yet well understood, angiomotin is thought to serve as a scaffold for MST1/2 and LATS1/2, and to directly bind and inhibit YAP1. Angiomotin is also speculated to bind and activate merlin, and thereby promote the binding of merlin to LATS1/2 [40].

### 3.2. NF2 Inactivation

*NF2* was shown more than two decades ago to constitute the target gene located within the 22q12 chromosomal region that is very frequently deleted in MM cells [41,42,43]. In fact, an estimated 40–50% of MM cases harbor an *NF2*-inactivating mutation or deletion, resulting in bi-allelic loss of function. *NF2* gene rearrangements have also been found to be frequently associated with MM. Of these, gene fusions have been demonstrated to be mutually exclusive with other types of mutations (e.g., point mutations) [8]. 

Merlin can also be inactivated via other mechanisms, such as the increased expression of protein kinase C-potentiated phosphatase inhibitor of 17 kDa (CPI-17) [44]. In particular, CPI-17 has been shown to inhibit MYPT-1-PP1δ, which in turn dephosphorylates merlin Ser518, thereby inactivating merlin to induce neoplastic cell transformations in vitro [45]. A previous study showed that a carboxyl-terminus *NF2* (isoform 2) splicing variant does not exert any tumor suppressive activity [46], suggesting that functional merlin inactivation might also be caused by the expression of such variants [44]; however, more recent studies conversely reported that both merlin isoforms 1 and 2 act as tumor suppressors [47]. Merlin inactivation may also be caused via the upregulated expression of potential *NF2-*targeting microRNAs, such as hsa-miR-885-3p [48], although further research is required to elucidate how such mechanisms mediate MM pathogenesis. 

Biologically, merlin re-expression has been confirmed to markedly inhibit the motility, spread, and invasiveness of *NF2*-deficient MM cells [49], whereas conversely, siRNA-mediated merlin silencing has been shown to result in the enhanced spreading and invasion of mouse embryonic fibroblasts (MEFs). Specifically, merlin expression attenuates the phosphorylation of focal adhesion kinase (FAK) at the critical Tyr397 phosphorylation site, and thereby disrupts the interaction between FAK and its binding partners, Src and p85, the regulatory subunit of phosphatidylinositol-3-kinase (PI3K) [49]. These findings suggested that merlin inactivation is likely related, at least in part, to the upregulation of FAK activity.

As *NF2* mutations are frequently detected in MM cases, genetically-engineered *Nf2*-knockout mouse models have been developed to evaluate the impact of *NF2* inactivation on MM pathogenesis. Asbestos-exposed *Nf2* (+/−) knockout mice have resultantly been shown to exhibit markedly accelerated MM tumor formation compared to their asbestos-treated wild-type littermates [50,51]. Furthermore, loss of the wild-type *Nf2* allele, leading to biallelic inactivation, was observed in all and 50% of asbestos-induced MMs from *Nf2* (+/−) and wild-type mice, respectively [51]. Similar to human MMs, the tumors developed by the *Nf2* (+/−) mice exhibited frequent homologous deletions of the *Cdkn2a/Arf* locus and adjacent *Cdkn2b* tumor-suppressor gene, as well as reciprocal inactivation of *Tp53* in a subset of tumors that retained the *Arf* locus [51]. Moreover, conditional (mesothelium-specific) *Nf2*, *Ink4a/Arf*, and/or *Tp53* knockout mouse models have also been shown to exhibit an increased incidence of MM development [52]. 

Notably, the underphosphorylated form of merlin has been shown to translocate to the nucleus, bind to the E3 ubiquitin ligase CRL4^DCAF1^, and inhibit the capacity of CRL4^DCAF1^ to ubiquitinate its target proteins; thus, merlin likely also functions as a negative regulator of CRL4^DCAF1^ [53]. Accordingly, merlin has been confirmed to exert CRL4^DCAF1^-mediated tumor-suppressive activity in both MM and a MeT-5A immortalized mesothelial-cell lines [53]. As CRL4^DCAF1^ directly binds to LATS1/2 to direct their conjugation to ubiquitin [54], it is likely that in merlin-deficient cells, de-repressed CRL4^DCAF1^ promotes LATS1/2 ubiquitination and degradation, and thus activates YAP1 by suppressing YAP1 phosphorylation.

Merlin also exhibits a cell-density-dependent, albeit Hippo-independent tumor-suppression activity via its downstream target, Lin28B (a *let-7* microRNA inhibitor) [55]. Specifically, merlin is phosphorylated and thus does not bind to Lin28B at low cell densities; thus, Lin28B is able to enter the nucleus and both bind to and inhibit the maturation of pri-*let-7* microRNAs. As *let-7* microRNAs act as tumor suppressors by silencing the expression of critical oncogenes such as *MYC* and *RAS,* inhibiting pri-*let-7* maturation promotes cell growth. 

Finally, *TRAF7*, which encodes an atypical member of the tumor necrosis factor (TNF) receptor-associated factor family, is occasionally mutated in MM. A previous study showed that four of five identified MM-associated *TRAF7* mutations were mutually exclusive with *NF2* mutations [8]. Notably, a similar pattern of exclusivity has been observed in *TRAF7*-mutated meningioma cases, which also frequently exhibit *NF2* mutations [56]. These observations suggest that TRAF7 and merlin may function in a common signaling pathway.

### 3.3. Hippo Pathway Component Inactivation

Hippo pathway dysregulation has been indicated in a broad range of human carcinoma types including lung, colorectal, ovarian, and liver cancers. Accordingly, the Hippo pathway has been investigated in these contexts as a potential therapeutic target [57,58,59,60]; however, these commonly occurring tumors only rarely exhibit genetic mutations in Hippo pathway components. In contrast, MM appears to be the only tumor type that is frequently associated with Hippo pathway mutations. In addition to *NF2* mutations, genetic *LATS2* inactivation is observed in MM cases, such that *LATS2* inactivation, and its effects tumor suppression, were initially identified in seven of 20 MM cell lines analyzed in vitro [61]. Similarly, 11% of 61 MPM primary cultures were found to harbor point mutations and/or large exon deletions that inactivated *LATS2* [62]. Together, these findings indicate that *NF2* and *LATS2* mutations can be coincident in a given MM tumor. A homozygous *SAV1* deletion was also detected in an MM cell line [61], and one of 16 analyzed MM cell lines that were subjected to whole exome sequencing was previously shown to harbor a LATS1-inactivating *LATS1-PSEN1* fusion gene [63]. Finally, a comprehensive genomic analysis of MM samples revealed frequent copy number loss among various Hippo pathway genes, including *MST1* and *LATS1* [8].

MM cells frequently exhibit downregulation of the LIM-domain protein AJUBA, which is a LATS2 binding partner [64]. Consistent with this finding, MM cell lines in which AJUBA is downregulated have been shown to exhibit higher levels of YAP1 dephosphorylation, whereas conversely, transducing MM cells with AJUBA has been demonstrated to significantly suppress YAP1 activity. Together, these data indicate that AJUBA negatively regulates YAP1 via its interactions with LATS1/2 [64]. In contrast, AJUBA has been suggested to exert oncogenic effects and to regulate the Hippo pathway in the context of other malignancies [65]; thus, the effects of AJUBA family LIM proteins on tumor development and progression require further clarification [66]. 

## 4. YAP1/TAZ

### 4.1. YAP1/TAZ Activation in MM Cells

As discussed, merlin-Hippo signaling inactivation leads to constitutive YAP1/TAZ activation. Moreover, YAP1 expression was observed in more than 70% of the analyzed primary MM tissues, with most positive cases showing greater YAP1-staining in the nucleus than the cytoplasm [61].

Gene amplification comprises one of several mechanisms of oncogene activation; accordingly, the chromosome 11q22 region where *YAP1* is located has been shown to be amplified in a variety of human malignances [67]. In addition to *YAP1*, this region includes a cluster of matrix metalloproteinase (MMP) genes, two members of the BIRC family of caspase inhibitors (*BIRC2* and *BIRC3*), and the progesterone receptor (*PGR*) [67]. Although 11q22 amplification is an infrequent event in MMs, *YAP1* amplification has been detected in a subset of MM cases, and YAP1 has been shown to be an important effector of MM cell proliferation in vitro [68]. 

### 4.2. YAP1/TAZ Enhance the Transcription of Pro-Oncogenic Genes

Activation of the YAP1 transcriptional coactivator has been shown to induce the transcription of multiple cancer-promoting genes in MM cells (Figure 1), including cell cycle promoting genes such as cyclin D1 (*CCND1*) and forkhead box M1 (*FOXM1*) [69], connective tissue growth factor (*CTGF*) [70], and phospholipase-C beta 4 (*PLCB4*) [71]. 

**Figure 2:**
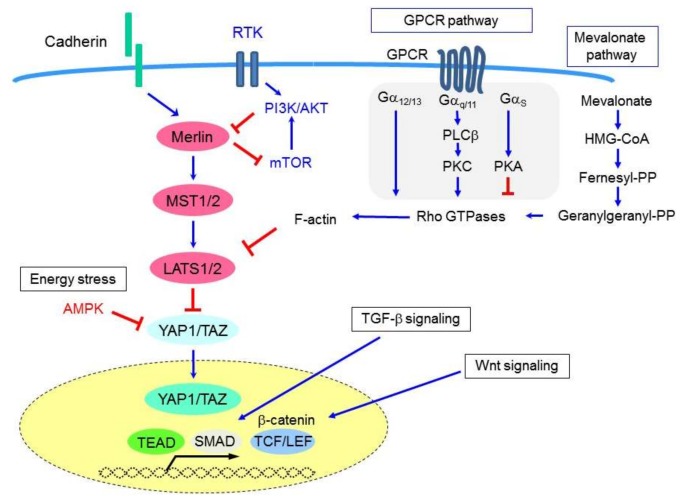
Signaling cascades and molecules that affect Hippo pathway signaling and YAP1/TAZ activation in MM cells. In addition to cell-to-cell adhesion status and RTK signaling, the Hippo pathway is also regulated by multiple signals including from the GPCR pathway, mevalonate pathway, and energy stress. Upon the activation of other intracellular signaling such as through TGF-β and Wnt signaling pathways, the transcription of YAP1/TAZ-target genes is further enhanced, which induces more aggressive malignant phenotypes of MM cells including cell proliferation, invasion, and EMT.

CTGF (or CCN2) is a member of the CCN protein family, which includes six members of secretory extracellular matrix-associated matricellular proteins. CTGF is a cysteine-rich protein that regulates cell-extracellular matrix (ECM) interactions and exerts multicellular functions including the regulation of cell proliferation, adhesion, migration, fibrosis, and inflammation. Notably, CTGF expression has been shown to be associated with abundant extracellular matrix formation in MM tissues [70]. Furthermore, CTGF expression was found to be significantly enhanced in MM cells in response to Hippo signaling inactivation, TGF-β stimulation [70], and/or β-catenin-TCF-LEF signaling [72] (Figure 2). CTGF has also been suggested to be highly expressed in sarcomatoid-type MMs and has been shown to mediate the epithelial-mesenchymal transition (EMT) in MM [72]. Similarly, another CCN family member, CYR61 (or CCN1), is also a well-known YAP1 target; however, its role in MM development and progression has not yet been determined.

The Hippo pathway is also regulated by G-protein-coupled receptor (GPCR) signaling [73] (Figure 2). Phospholipase-C, the downstream effector of Gαq signaling, is required to enable inositol 1,4,5-triphosphate and diacylglycerol to be formed from phosphatidylinositol 4,5-biphosphate, and thus also for signal transduction. *PLCB4* (which encodes phospholipase-C β4) was shown to be a YAP1-target gene [71], as inducing *PLCB4* knockdown attenuated the growth of YAP1-transduced immortalized mesothelial cells, and YAP1-active (but not YAP1-non-active) MM cells [71]. 

Similarly, uveal melanoma has been reported to frequently harbor activating mutations in *GNAQ* and *GNA11*. These genes encode two highly homologous Gα_q/11_ heterotrimeric G-protein α subunits, and their activation has been shown to activate YAP1 [74,75]. *PLCB4* is also occasionally mutated in uveal melanoma [76], suggesting that a positive feedback loop may link YAP1 activation with GPCR signaling in a subset of human malignancies. Notably, uveal melanomas are also frequently associated with *BAP1* mutations, in the same manner as MM [10,77,78,79,80].

*BAP1* encodes a protein that regulates transcription, histone modification, DNA repair [81], and Ca^2+^ flux from the endoplasmic reticulum to mitochondria [82]; thus, there may be an as yet unknown functional relationship between the YAP1 activation and BAP1 inactivation that occurs during the development of both of these tumor types. 

### 4.3. YAP1/TAZ Enhance the Tumorigenicity of Immortalized Mesothelial Cells

Cancer develops via the accumulation of genetic and epigenetic alterations that promote malignant cell phenotypes [83]. Although the processes by which these occur during MM pathogenesis are not well understood, changes to critical MM-associated tumor suppressor genes are thought to confer one or more malignant phenotypes to normal mesothelial cells [84].

Immortalized mesothelial cells that were generated via the transduction of human papilloma virus *E6/E7* and *hTERT* genes were used to investigate the mechanisms underlying the effects of YAP1 activation on malignancy. Treatment of the cells with exogenously transduced wild-type *YAP1,* and even more so, with an activated mutant *YAP1 S127A,* stimulated the mesothelial cells to form mesothelioma-like tumors. The effect was more obvious when the immortalized cells were inoculated into the thoracic cavities of nude mice [71]. 

## 5. Diagnosis and Prognostic/Predictive Biomarkers Based on Hippo Pathway Dysregulation

As *NF2* is frequently inactivated in MM cells, its deletion status has been investigated as a potential MM diagnostic marker. Such markers are needed to aid the diagnosis of MPM in the clinical setting, because it is sometimes difficult to differentiate between MPM and other malignancies (such as lung cancer and sarcoma) that develop in the thoracic cavity, and/or reactive mesothelial hyperplasia (RMH), which is a benign condition that essentially requires no further treatment. Adding genetic *NF2* screening to the standard methods of using fluorescence in situ hybridization (FISH) techniques to identify *CDKN2A* (*p16^Ink4a^/p14^Arf^)* deletions did not significantly improve the sensitivity or specificity of MM diagnosis. Currently, *CDKN2A* FISH and BAP1 immuno-histochemical staining are considered to be the most effective methods of differentiating between MMs, other malignancies, and RMH [85]. A more convenient immunohistochemical analysis method for detecting the product of the *MTAP* gene, (which is adjacent to and often co-deleted with *CDKN2A)*, has also been recently shown to be useful in diagnosing MM [85]. 

Both a low level of merlin expression and a high survivin labeling index have been shown to be indicators of a poor prognosis in patients with MPM [86], suggesting their potential as representing possible prognostic markers for MM. Likewise, a combination of homozygous *CDKN2A* deletions and hemizygous *NF2* loss in peritoneal mesotheliomas has been shown to be an independent negative prognostic factor for both PFS and OS [87].

Recent studies have shown that a FAK inhibitor termed VS-4718 inhibits proliferation and induces apoptosis in MM cells that lack merlin expression [88]. This preferential effect of VS-4718 in merlin-deficient cells suggests that merlin may constitute a potential predictive biomarker for the enhanced response of MM cells to VS-4718 treatment. However, a phase-II (registration-directed, double-blind, placebo-controlled study (COMMAND)) trial of another selective FAK inhibitor, VS-6063 (or defactinib), for use in patients with mesothelioma was recently halted, owing to a lack of any observed benefits (despite correcting for merlin deficiency). Thus, whether FAK inhibitors can provide a clinical benefit for patients with merlin-negative MM cells remains unclear. Notably, another study recently reported that E-cadherin expression was correlated with resistance of the merlin-negative MM cells to FAK inhibitor treatment [89].

## 6. Therapeutic Applications Based on Hippo Pathway Dysregulation

**Figure 3:**
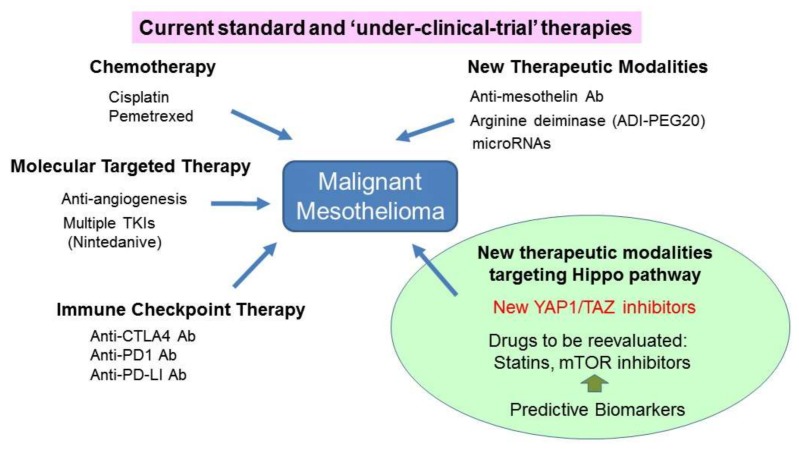
Overall concept of the current, and “under-clinical-trial” therapies available to patients with malignant mesothelioma (MM), as well as potential novel therapeutic modalities that target the Hippo pathway. Ab, antibody; TKIs, tyrosine kinase inhibitors.

Given that MM is highly refractory to current therapeutic modalities, new drugs, and novel therapeutic strategies based on the molecular and cellular characteristics of MM are urgently required to improve patient outcomes. Based upon the current literature, targeting the Hippo pathway is likely to be a very promising therapeutic approach for this disease (Figure 3) [90].

### 6.1. Targeting YAP1/TAZ

The significant impact of Hippo pathway dysregulation and constitutive YAP1/TAZ activation on MM cells renders these events highly attractive as molecular targets for the development of novel therapeutic approaches to MM. Restoring upstream regulatory Hippo pathway components would restore Hippo pathway function; however, reintroducing tumor suppressor gene expression and activation to whole cells in tumor tissues is likely to be technically very difficult. 

An alternative approach is to block YAP1/TAZ interactions with their target transcription factors. For example, TEADs are thought to be the factors primarily expressed and involved in the prooncogenic functions of YAP1/TAZ in MM cells; thus, the disruption of YAP1/TAZ and TEAD interaction may represent a very promising approach. In this regard, several molecules have been developed and tested in an attempt to develop new therapeutic agents against human malignancies. The first small molecule shown to function as a YAP1-TEAD binding inhibitor was verteporfin (Visudyne), which is used clinically as a photosensitizer in photodynamic therapy for neovascular macular degeneration [91]. Notably, verteporfin has been shown not to require light activation to inhibit the growth and motility of cultured breast-cancer cells [91], and has been demonstrated to inhibit other human cancer-cell types including ovarian, hepatoma, and glioblastoma cancer cells in vitro [92]. Moreover, verteporfin treatment was also shown to suppress YAP1 activity along with the viability, invasion, and tumor sphere formation of MM cell lines in vitro [62,93]. 

Other YAP1-TEAD inhibitors have also been developed including the bioengineer-designed, cyclic YAP1-like peptides (17 mer), which have been demonstrated to interrupt the YAP-TEAD interaction [94]. Mammalian vestigial-like 4 (VGLL4) was also previously identified as a natural YAP1 antagonist that binds to TEADs via its Tondu (TDU) domains, such that the VGLL4 TDU region is sufficient to inhibit YAP1 activity [95,96]. Based on these observations, a synthetic peptide (48 mer) that mimicks the VGLL4 TDU domain (Super-TDU) was synthesized and shown to suppress gastric and lung cancer cells by competing with YAP1 to bind to TEADs [95]. Recently, VGLL4 was also shown to target a TCF4-TEAD4 complex, suggesting that it may also function as a regulator of Wnt/β-catenin signaling [97]. In support of this mechanism, Super-TDU treatment has been shown to reduce the number of colorectal adenomas exhibited by APC^min/−^ mice [97]. In addition, TIAM1, a component of the Wnt-regulated destruction complex, has recently been shown to antagonize TAZ and YAP1 in colorectal cancer cells by promoting TAZ cytoplasmic degradation and suppressing YAP1/TAZ interactions with TEADs [98]. As MM cells have also been reported to exhibit Wnt signaling activation [99], these inhibitors should be further investigated to determine their effects on both YAP1/TAZ and Wnt activated MM cells. 

### 6.2. Targeting YAP1 Using Metabolic-Disorder Drugs

The cellular metabolic status, including cellular responses to glucose deprivation (energy stress) and the mevalonate cascade, has been shown to be linked to Hippo signaling [100]. Glucose deprivation reduces cellular ATP levels and induces activation of the AMP-dependent protein kinase (AMPK), which is a sensor of cellular energy stress. AMPK has been found to attenuate YAP1 activity via multiple mechanisms, such as reducing nuclear YAP1 levels and inhibiting YAP1-TEAD interactions [101,102,103]. The widely-used anti-diabetic drug metformin, which reduces blood glucose levels and activates AMPK, has previously been shown to inhibit YAP1. Moreover, it has been suggested that metformin functions as an anticancer agent, predominantly via mTOR pathway inhibition; thus, its administration has been reported to be associated with improved survival in the treatment of patients with diabetes plus several types of cancers [104]. However, a retrospective study of 300 patients with type 2 diabetes and MPM did not shown any improvement to patient survival [105]. Meanwhile, metformin has been recently shown to inhibit MM cell proliferation when administered in combination with nutlin-3a (an inhibitor of ubiquitin-mediated p53 degradation) [106]. 

Statins (i.e., inhibitors of the rate-limiting enzyme (HMG-CoA reductase) of the mevalonate cholesterol biosynthesis pathway) are currently used to treat hypercholesterolemia and prevent cardiovascular diseases. They have also been found to exert anticancer effects in many cancer types, with lovastatin in particular having been shown to induce apoptosis in human MM cell lines [107]. Statins have also been found to be effective against MM in vivo when combined with doxorubicin chemotherapy, likely by reducing the ability of the MM cells to acquire resistance to doxorubicin treatment [108]. In contrast, both a mouse model and human cohort study of 1738 patients with a history of asbestos exposure reported that statins do not moderate mesothelioma development or progression [109]. Although the putative preventive and therapeutic effects of statins in the treatment of various cancers, including mesothelioma, require further investigation, their effects on the mevalonate pathway have been confirmed to control YAP1/TAZ activity [110], such that statin treatment inhibits both YAP1/TAZ nuclear localization and transcriptional responses [110]. Mechanistically, the geranylgeranyl pyrophosphate produced by the mevalonate cascade is required for the activation of Rho GTPases that, in turn, activate YAP1/TAZ, likely via actin cytoskeleton rearrangements [110,111]. Consistent with these data, statins have been demonstrated to exert greater antiproliferative activity in MM cells that exhibit Hippo pathway inactivation [112]. Conversely, they exert a weaker inhibitory effect in MM cells that also harbor *BAP1* mutations [112]; thus, the effectiveness of statin treatments in MM cells appears to vary dependent upon the cellular genetic/epigenetic background.

### 6.3. Targeting Molecules Activated by Merlin Deficiency

Merlin regulates mitogenic signaling by suppressing mTORC1 in MM cells, as demonstrated by the fact that merlin-deficient MM cells have been shown to be selectively sensitive to the growth-inhibiting effects of the allosteric mTOR inhibitor, rapamycin [113]. Although the mechanisms by which merlin suppresses mTOR signaling remain elusive, *NF2* inactivation has been found to confer sensitivity to rapalogs in bladder cancer [114], suggesting that mTORC1 signaling likely sustains the expansion of merlin-deficient cancer cells. 

A phase-II study of an mTOR inhibitor everolimus was conducted in advanced MPM, in which a total of 59 patients were evaluable for 4-month PFS [115]. The results of the trial suggested that everolimus had limited clinical activity, and that its use as a single-agent therapy for advanced MPM was not warranted. In comparison, a recent phase-I study of the dual class-I PI3K and mTOR kinase inhibitor apitolisib (GDC-0980) showed a partial response in patients with pleural and peritoneal mesothelioma [116]. 

As discussed, merlin loss (at least in part) drives tumorigenesis via E3 ubiquitin ligase CRL4^DCAF1^ activation and subsequent LATS1/2 inhibition. MLN4924, a NEDD8-activating enzyme (NAE) inhibitor, has been shown to suppress CRL4^DCAF1^ and attenuate YAP1 activation in *NF2*-mutant tumor cells [117]. Although MLN4924 alone did not exhibit significant preclinical activity, administering MLN4924 with the mTOR/PI3K inhibitor GCD-0980 suppressed the growth of *NF2*-mutant tumor cells in vitro as well as in mouse and patient-derived xenografts [117]. 

### 6.4. Targeting CTGF

*CTGF* is a well-known YAP1 target gene that encodes an ECM-associated protein. Deregulated CTGF expression has been observed in many types of human malignancies including MM, and has been shown to be associated with increased cell proliferation, drug resistance, angiogenesis, adhesion/migration, and metastasis [118]. Although *CTGF* transcription is induced by YAP1 activation, its expression in MM cells has also been demonstrated to be enhanced by both TGF-β and Wnt signaling [70,72]. 

CTGF levels are also elevated in patients with fibrotic lung disease including idiopathic pulmonary fibrosis (IPF). The human anti-CTGF monoclonal antibody pamrevlumab (FG-3019) is currently undergoing clinical testing for IPF and other indications [119]. As FG-3019 has been shown to be effective against high-grade serous ovarian cancer [120], it should also be investigated as a potential therapeutic agent for MM. Furthermore, a murine pancreatic ductal adenocarcinoma (PDA) model indicated that the efficacy of FG-3019 was increased when it was administered in combination with gemcitabine [121]. As both PDA and desmoplastic-type MM (a rare MM subtype characterized by a very poor patient prognosis) are characterized by excess ECM deposition, they may comprise good candidates for CTGF inhibitor treatment. 

### 6.5. Extracellular Stimuli

Numerous extracellular stimuli regulate the Hippo pathway including cell-cell adhesion, cell-ECM contact, mechanical stresses (e.g., stiffness), and soluble factors [37]. 

Cell attachment to the ECM regulates Hippo pathway activity. During detachment from the ECM, cells normally undergo anoikis (programmed cell death), which has been shown (in non-transformed cells) to be mediated by YAP1 inactivation. Conversely, expression of constitutively active YAP1 has been found to promote the survival of detached cells [122]. Characteristically, mesothelial cells are thought to be relatively anoikis-resistant. When serosal injury occurs on the mesothelium, mesothelial cells not only migrate onto the wound surface from the wound edge but also detach from opposing surfaces and migrate to a distant site, where they settle on the wound surface to initiate wound repair [123]. Although it is unclear how YAP1/TAZ is involved in these mesothelial repair processes, the anoikis-resistant phenotype characteristic of MM cells may be innate and/or enhanced by merlin-Hippo pathway inactivation.

As discussed, GPCR signaling regulates the Hippo pathway, such that many mitogenic hormones and growth factors act via GPCRs to induce cell proliferation [37]. For example, ligand signals such as lysophosphatidic acid (LPA), sphingosine-1-phosphate (S1P), and estrogen can activate YAP1/TAZ through GPCRs coupled to Gα_12/13_ or Gα_q/11_. In addition, ligand signals such as epinephrine and glucagon can repress YAP1/TAZ activity through Gαs-coupled GPCRs and protein kinase A (PKA). Targeting GPCRs is now recognized as promising therapeutic approach for cancer [124] that has been evaluated in clinical trials. One such agent is sonepcizumab, a humanized monoclonal antibody directed at S1P, for treatment of metastatic renal cell carcinoma [125]. With regard to MM, LPA was previously shown to stimulate the proliferation and motility of MPM cells [126]. Although it remains unclear how these soluble factors influence MM cell growth and progression, and/or whether they influence YAP1/TAZ activity in MM cells, recurrent GRCR mutations (including *GRM3*, *GPR149*, and *GPR98*) have also been identified in MM cells [8]. These data support the need for further study to clarify the association between GPCR signaling and YAP1/TAZ activation in MM cells. 

### 6.6. Other Possible Therapeutic MM Strategies

The two major families of kinases in the Hippo pathway, MST1/2 and LATS1/2, may also be promising therapeutic targets for MM, owing to their role in directing the phosphorylation and cytoplasmic sequestration of YAP1/TAZ. Thus, Hippo pathway agonists that activate MST1/2 and LATS1/2 kinases and inhibit YAP1 activation in cancer cells have been identified as candidate therapeutic agents [127]. For example, a small molecular compound termed C19 was recently shown to induce MST1/2 and LATS1/2 phosphorylation, and thereby stimulate TAZ degradation [128]. 

Several other distinct small molecules, including dasatinib, pazopanib, and ivermectin, have also been identified to inhibit YAP1/TAZ by drug screening using human cancer cell lines [129,130]. Dasatinib, a multi-targeted tyrosine kinase inhibitor against BCR/ABL, the Src family kinases, and PDGFRβ, was reported to suppress the proliferation, migration, and invasion of MM cells in vitro [131], and enhance pemetrexed-induced cytotoxicity [132]. Src phosphorylation was also shown to be suppressed by dasatinib treatment in surgically removed MM tissues [133]. Notably, however, the administration of dasatinib as a single-agent was not effective as a second-line treatment for MM during a recent phase II trial [16]; likewise, administration of dasatinib as a neoadjuvant treatment was not shown to confer any benefit to patients with MPM. In contrast, a recent study reported the ability of the anti-parasitic agent ivermectin to inhibit YAP1, and thereby exert significant effects on gastric cancer [134], hepatocellular carcinoma, and MM cells [130]. 

Additionally, a recent study showed that a TEAD4 splicing variant (TEDA4-S, in which exon 3 is skipped by the tumor suppressor RBM4 protein) acts as a dominant negative TEAD4 isoform, and thereby both attenuates YAP activity and inhibits tumor-cell proliferation [135]. It is possible that splicing dysregulation that affects other Hippo pathway genes may also mediate MM pathogenesis; thus, this process should be further investigated to identify novel MM therapeutic targets [135].

Finally, the induction of synthetic lethality constitutes another promising therapeutic strategy. For example, the poly(ADP-ribose) polymerase (PARP) inhibitor olaparib induces enhanced cytotoxicity in breast cancer cells that harbor *BRCA1/2* mutations, as their endogenous DNA damage repair systems are suppressed by both BRCA1 inactivation and PARP inhibition. Notably, when PARP inhibitors have been used to treat MM cell lines in vitro, they unexpectedly exerted inhibitory effects on all analyzed cell lines, regardless of their *BAP1* mutation status [136]. In contrast (as discussed above), MM treatment with a FAK inhibitor was expected to induce synthetic lethality when combined with merlin deficiency [88], but failed in a clinical trial. Nevertheless, inducing synthetic lethality is a new approach in the context of MM research and should be further developed to identify novel candidate agents/genes that effectively induce a synthetic-lethal phenotype when combined with merlin-Hippo deficiency or *BAP1* inactivation. 

### 6.7. Rationale and Challenges of Targeting Hippo Signaling to Treat MM

To date, very few studies have investigated the effects of YAP1/TAZ-inhibition on MM either in vitro or in vivo; nevertheless, targeting YAP1/TAZ likely represents a very promising therapeutic strategy for patients with MM for several reasons. Firstly, in contrast to other human malignancies, MMs are frequently and occasionally associated with mutations in *NF2,* and genes encoding other components of the Hippo pathway, respectively, strongly suggesting that disruption of merlin-Hippo signaling is a key event during MM development and progression. Secondly, MMs also frequently exhibit YAP1/TAZ activation, which event has been previously shown to confer malignant phenotypes to mesothelial cells. Conversely, YAP1/TAZ suppression inhibits MM cell proliferation, migration, and invasion. Thirdly, MMs harbor a relatively small number of genetic changes, predominantly in tumor suppressor genes; however, as MM-associated mutations rarely affect targetable (e.g., kinase-encoding) oncogenes, new therapeutic targets and strategies are needed. Various Hippo pathway components appear to be promising targets for inhibiting protein-protein binding. Fourthly, although clear evidence for their therapeutic efficacy has not yet been demonstrated, some small molecules that are thought to impact Hippo pathway activation have been previously shown to inhibit MM cell proliferation and progression. As most of these were initially tested without regard to the merlin-Hippo pathway dysregulation status of the analyzed MM cells, they require careful reevaluation. Finally, the current literature suggests that a combination strategy, comprising YAP1/TAZ suppression and the inhibition of an additional relevant pathway, may be more effective in treating MM than any single strategy alone.

However, the potential that targeting YAP1/TAZ might also induce serious and unexpected adverse effects in patients should be considered when pursuing this strategy, as the Hippo pathway is essential for various aspects of normal cell functions. Thus, possible side effects for any agents or strategies targeting the Hippo pathway as described above should be carefully evaluated in both preclinical studies and clinical trials.

Additionally, although immunohistochemical analyses of merlin and YAP1/TAZ expression are often used to evaluate the merlin-Hippo pathway status in MM cells, the current methodologies and antibodies are neither very sensitive nor specific. More precise and rapid assays, along with more effective MM biomarkers, are needed to accurately determine which MM cases are associated with activated YAP1/TAZ (e.g., for drug development or patient screening) in the context of both laboratory and clinical trial-based research. 

## 7. Conclusions

MM is a highly aggressive disease, which, along with pancreatic and bile-duct cancer, is recognized as one of the most formidable cancer types. The development of new therapies for this disease has lagged behind that achieved for other common malignancies, so that only a limited number of therapeutic strategies are currently available to patients. Although immune-checkpoint and anti-angiogenesis therapies have shown some promising results during preliminary testing, new and effective therapeutic modalities are still urgently needed [137]. The current literature supports the development of a novel approach based on the Hippo pathway dysregulation observed in MM cells as likely to be effective against MM. However, additional studies are required to elucidate and characterize the mechanisms underlying, and the impact of Hippo pathway inactivation, on the pathogenesis of this disease. 

## Abbreviations

Ab	antibody
AMPK	AMP-dependent protein kinase
ASS1	arginosuccinate synthetase-1
BAP1	BRCA1-associated protein 1
BIRC	baculoviral IPA repeat containing
CCDN1	cyclin D1
CCN	CYR61, CTGF, NOV
CDKN2A	cyclin dependent kinase inhibitor 2A
CTGF	connective tissue growth factor
CPI-17	C-potentiated phosphatase inhibitor
CRL	cullin-RING E3 ubiquitin ligase
DCAF1	DDB1 and CUL4 associated factor 1
DNMT	DNA methyltransferase
ECM	extracellular matrix
EGFR	epidermal growth factor receptor
EMT	epithelial-mesenchymal transition
ERM	ezrin/radixin/moesin
EZH2	enhancer of Zeste homolog 2
FAK	focal adhesion kinase
FGFR	fibroblast growth factor receptor
FISH	fluorescence in situ hybridization
FOXM1	forkhead box M1
GPCR	G-protein-coupled receptor
HDAC	histone deacetylase
HMG-CoA	hydroxymethylglutaryl-CoA
IPF	idiopathic pulmonary fibrosis
LATS	large tumor suppressor kinase
LEF	lymphoid enhancer-binding factor
MM	malignant mesothelioma
MPM	malignant pleural mesothelioma
MST	mammalian sterile 20-like protein kinase
MTAP	methylthioadenosine phosphorylase
mTOR	mammalian/mechanistic target of rapamycin
mTORC1	mTOR complex 1
MYPT-1-PP1δ	myosin phosphatase targeting subunit 1-protein phosphatase 1δ
NF2	neurofibromatosis type 2
OS	overall survival
PAK	p21-acivated kinase
PARP	poly(ADP-ribose) polymerase
PDA	pancreatic ductal adenocarcinoma
PDGFR	platelet-derived growth factor receptor
PD1	programmed cell death 1
PD-L1	programmed cell death ligand 1
PFS	progression free survival
PI3K	phosphoinositide 3-kinase
PKA	protein kinase A
PKC	protein kinase C
PLCB4	phospholipase-C β 4
RTK	receptor tyrosine kinase
RMH	reactive mesothelial hyperplasia
TAZ	transcriptional coactivator with PDZ-binding motif
TCF	transcription factor
TEAD	TEA domain transcription factor
TERT	telomerase reverse transcriptase
TGF-β	transforming growth factor β
TIAM1	T-cell lymphoma invasion and metastasis 1
TKI	tyrosine kinase inhibitor
TRAF7	TNF receptor associated factor 7
VEGFR	vascular endothelial growth factor receptor
VGLL4	vestigial-like 4
YAP1	Yes-associated protein 1
